# Enhanced Recovery After Surgery Versus Conventional Care in Colorectal Surgery: A Systematic Review of Randomized Controlled Trials

**DOI:** 10.7759/cureus.88007

**Published:** 2025-07-15

**Authors:** Dixon Osilli, Muhammad Muaz Loon, Nouman Anthony, Simran James, Sergio Rodrigo Oliveira Souza Lima, Muhammad Ali

**Affiliations:** 1 General Surgery, Queen's Hospital, Romford, GBR; 2 General Surgery, Barking, Havering and Redbridge University Hospitals National Health Service (NHS) Trust, London, GBR; 3 General Medicine, Rehman Medical Institute, Peshawar, PAK; 4 Gynaecology, Rehman Medical Institute, Peshawar, PAK; 5 Plastic Surgery, Bahia Hospital, Salvador, BRA; 6 General Surgery, Nishtar Medical University, Multan, PAK

**Keywords:** colorectal surgery, enhanced recovery after surgery, eras, fast-track recovery, length of hospital stay, perioperative care, postoperative morbidity, randomized controlled trials, systematic review

## Abstract

Enhanced Recovery After Surgery (ERAS) protocols have gained prominence in colorectal surgery as a means to improve patient outcomes and reduce postoperative recovery time. This systematic review evaluated the effectiveness of ERAS protocols compared to conventional perioperative care, focusing on postoperative morbidity and length of hospital stay (LOS). A comprehensive literature search was conducted across PubMed, Scopus, Web of Science, and Cochrane CENTRAL for randomized controlled trials (RCTs) published up to April 2024. Studies included adult patients undergoing elective colorectal surgery and reported on LOS and postoperative complications. Three RCTs met the inclusion criteria. Two trials demonstrated statistically significant reductions in LOS in ERAS groups compared to controls (p = 0.001 and p = 0.021), while the third reported no significant difference but affirmed ERAS safety. None of the studies showed increased morbidity, readmission rates, or mortality associated with ERAS protocols. ERAS protocols are a safe and effective alternative to conventional perioperative care in colorectal surgery. They are consistently associated with reduced hospital stay and comparable complication rates, supporting their broader implementation to enhance recovery and healthcare efficiency.

## Introduction and background

Colorectal surgery is among the most commonly performed major abdominal procedures worldwide, often associated with significant postoperative morbidity and prolonged hospital stay [1]. Conventional perioperative care typically involves extended fasting periods, delayed mobilization, liberal use of opioids, and prolonged hospitalizations, which can delay recovery and increase healthcare costs [2]. In recent years, the evolution of perioperative management strategies has prompted the development and adoption of Enhanced Recovery After Surgery (ERAS) protocols, a multimodal approach aimed at optimizing patient outcomes through evidence-based interventions [3].

ERAS programs, first introduced in colorectal surgery, integrate several perioperative elements such as preoperative counseling, early oral feeding, minimally invasive techniques, multimodal analgesia, and early mobilization. These protocols are designed not only to attenuate the physiological stress response to surgery but also to accelerate functional recovery, reduce complication rates, and shorten length of hospital stay (LOS) [4]. Numerous studies have reported the benefits of ERAS in various surgical disciplines, particularly in colorectal surgery, where the approach is now increasingly being integrated into routine practice [5].

Despite the growing body of literature supporting ERAS, variations in implementation, patient populations, and hospital settings have led to inconsistent outcomes in some cases. Moreover, while systematic reviews and meta-analyses have broadly assessed ERAS protocols, there remains a need for a focused synthesis of randomized controlled trials (RCTs) that directly compare ERAS to conventional perioperative care in colorectal surgery. Evaluating such high-quality evidence is critical to understanding the actual impact of ERAS on clinical outcomes, particularly postoperative morbidity and LOS, which are key metrics of surgical success and healthcare efficiency. The objective of this systematic review is to evaluate and synthesize the evidence from RCTs comparing ERAS protocols to conventional perioperative care in patients undergoing colorectal surgery, with a focus on two primary outcomes: postoperative morbidity and LOS.

## Review

Materials and methods

Study Design and Reporting Guidelines

This systematic review was designed to evaluate the impact of ERAS protocols compared to conventional perioperative care in colorectal surgery, with a focus on postoperative morbidity and LOS. The review was conducted in accordance with the Preferred Reporting Items for Systematic Reviews and Meta-Analyses (PRISMA) 2020 guidelines [6], ensuring methodological transparency and reproducibility.

Eligibility Criteria (PICO Framework)

The eligibility criteria for this review were defined using the PICO framework [7]. The Population included adult patients undergoing elective colorectal surgery. The Intervention consisted of ERAS protocols, which typically incorporate preoperative counseling, early oral feeding, early mobilization, and multimodal, opioid-sparing analgesia. The Comparison group received conventional perioperative care without the structured implementation of ERAS protocols. The Outcomes of interest were primarily the LOS and postoperative morbidity, including complication rates and hospital readmissions. Only RCTs published in English were considered for inclusion. Studies were excluded if they were non-randomized, observational in design, case reports, reviews, editorials, or not focused on colorectal surgery, or if they failed to report on either LOS or morbidity outcomes.

Search Strategy

A comprehensive literature search was conducted across the following databases: PubMed, Scopus, Web of Science, and Cochrane Central Register of Controlled Trials (CENTRAL). The search included studies published up to April 2024. Keywords and MeSH terms used included "Enhanced Recovery After Surgery" OR "ERAS" OR "fast-track surgery" AND "colorectal surgery" OR "colonic resection" OR "rectal surgery" AND "randomized controlled trial" OR "RCT". Filters were applied to include only studies involving human subjects, written in English, and with full-text availability. Additional manual screening of reference lists from eligible articles and relevant reviews was performed to ensure comprehensive inclusion.

Study Selection and Data Extraction

After the removal of duplicates, titles and abstracts were screened independently. Full texts of potentially eligible studies were reviewed to confirm inclusion. For each selected RCT, the following data were extracted into a standardized table: author, year, study design, sample size, intervention and control descriptions, primary outcomes, and key results. Discrepancies during study selection or data extraction were resolved through discussion and consensus.

Risk of Bias Assessment

The Cochrane Risk of Bias 2.0 (RoB 2) tool [8] was used to assess the quality of each included RCT. This tool evaluates bias across five domains: (1) bias from the randomization process, (2) bias due to deviations from intended interventions, (3) bias due to missing outcome data, (4) bias in measurement of outcomes, and (5) bias in selection of the reported results. Each study was assigned an overall judgment of Low Risk, Some Concerns, or High Risk based on these criteria.

Data Synthesis

Due to the limited number of trials and heterogeneity in ERAS protocol elements, a qualitative synthesis was performed. Study outcomes, including LOS and morbidity rates, were compared narratively to identify consistent trends and differences between ERAS and conventional care across the included RCTs.

Results

Study Selection Process

**Figure 1:**
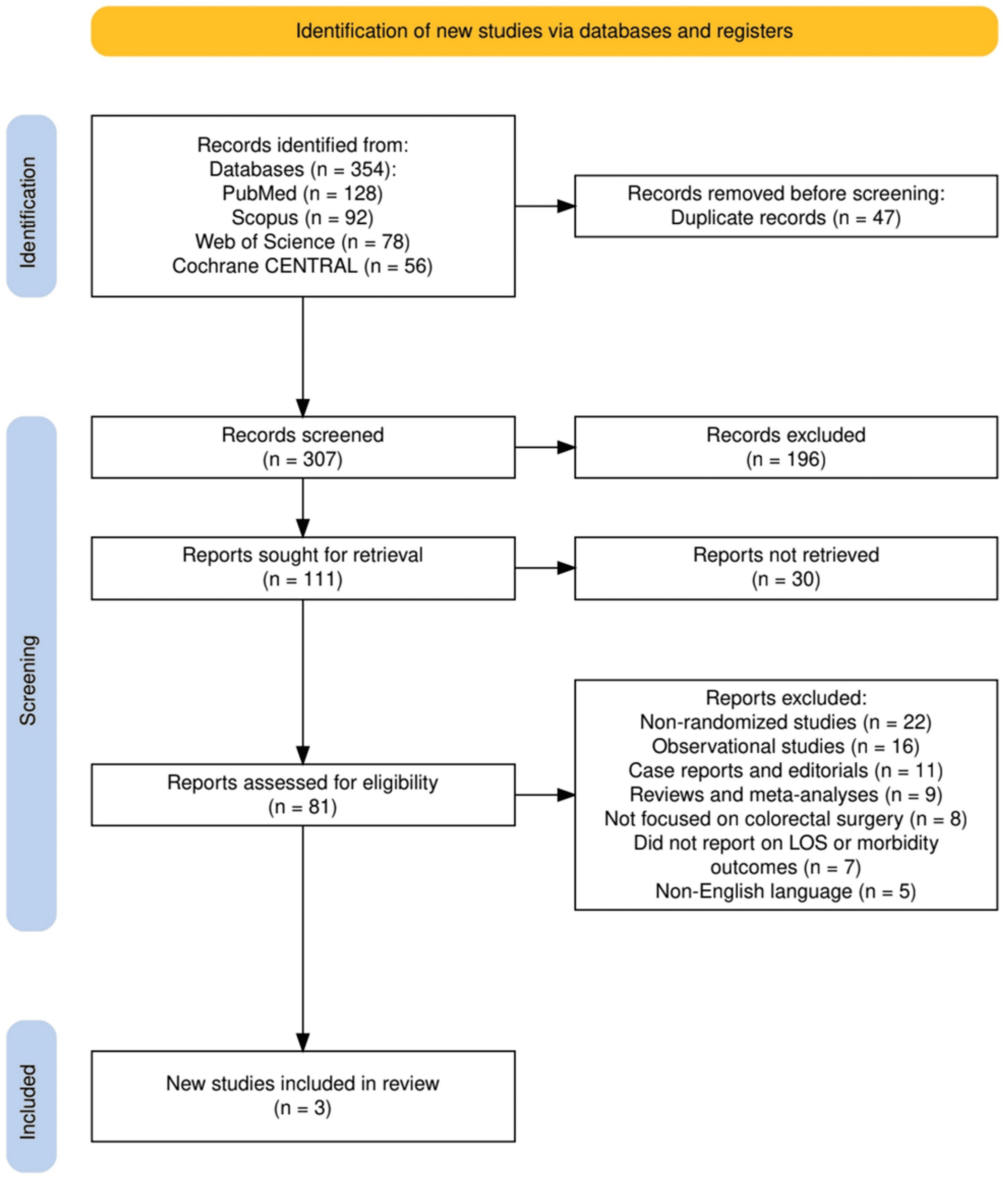
The PRISMA flowchart represents the study selection process. PRISMA: Preferred Reporting Items for Systematic reviews and Meta-Analyses; LOS: length of hospital stay

The study selection process followed the PRISMA 2020 guidelines and is detailed in Figure 1. A total of 354 records were identified through database searches: PubMed (128), Scopus (92), Web of Science (78), and Cochrane CENTRAL (56). After removing 47 duplicate records, 307 studies remained for title and abstract screening. Of these, 196 records were excluded based on relevance. A total of 111 full-text reports were sought for retrieval, but 30 could not be accessed. The remaining 81 full-text articles were assessed for eligibility. Following the predefined inclusion and exclusion criteria, 78 studies were excluded for reasons including being non-randomized (n = 22), observational in design (n = 16), case reports or editorials (n = 11), reviews or meta-analyses (n = 9), not focusing on colorectal surgery (n = 8), failing to report on LOS or morbidity (n = 7), or not published in English (n = 5). Ultimately, three RCTs met all inclusion criteria and were included in the final systematic review.

Characteristics of the Selected Studies

The characteristics of the three RCTs included in this review are summarized in Table 1. All studies compared ERAS protocols with conventional perioperative care in adult patients undergoing colorectal surgery. The interventions varied slightly in focus, ranging from full ERAS programs to early rehabilitation protocols, but commonly included elements such as early mobilization, oral feeding, and patient counseling. Sample sizes varied, with one large-scale cluster trial and two smaller single-center RCTs. Across all studies, the primary outcome was LOS, with postoperative morbidity as a key secondary outcome. Two studies reported statistically significant reductions in LOS in the ERAS groups compared to controls, while one study found no significant difference. Importantly, none of the trials showed a significant increase in complication rates, readmissions, or mortality associated with ERAS protocols.

**Table 1:** Summary of study characteristics, interventions, outcomes, and key findings from randomized controlled trials comparing ERAS protocols with conventional perioperative care. RCT: randomized controlled trial; ERAS: Enhanced Recovery After Surgery; LOS: length of hospital stay; QoL: quality of life; p: p-value (probability value used in statistical significance testing)

Study (author, year)	Study design	Sample size	Intervention description	Control description	Primary outcomes	Key results
Forsmo et al., 2016 [9]	RCT	324	ERAS protocol including pre- and postoperative counseling, early feeding, mobilization, and standardized discharge criteria	Conventional perioperative care	Total hospital stay	Median LOS: ERAS 5 days vs. control 8 days (p = 0.001); no significant difference in complications, readmissions, or mortality
Pagano et al., 2024 [10]	Stepped-wedge cluster RCT	ERAS: 1,337/control: 1,060	Region-wide ERAS implementation supported by audit & feedback and standardized training	Standard care prior to ERAS rollout	LOS without outliers (>94th percentile); complications	Mean LOS: ERAS 7.5 vs. control 8.5 days; adjusted reduction: -0.58 days (p = 0.021); no significant difference in complications
Lee et al., 2013 [11]	RCT	ERAS: 52/control: 46	Early rehabilitation after laparoscopic low anterior resection, including early mobilization and oral feeding	Conventional care with delayed mobilization and feeding	Recovery rate at 4 days; secondary: LOS, complications, QoL	No significant difference in LOS (7.5 vs. 8.0 days, p = 0.882); trend toward more complications in ERAS (not statistically significant); similar QoL and pain

Quality Assessment

The quality assessment of the included studies was conducted using the Cochrane RoB 2 tool, and the results are summarized in Table 2. One study was rated as having an overall low risk of bias, demonstrating clear randomization procedures, adherence to interventions, complete outcome reporting, and use of standard measurement methods. The second study, a cluster randomized trial, was judged to have some concerns primarily due to the potential for imbalance in the randomization process inherent to the stepped-wedge design, despite strong monitoring and low attrition. The third study also presented some concerns, particularly related to deviations from the intended intervention and measurement bias, as subjective outcomes like pain and quality of life were assessed without blinding. Overall, the quality of evidence across the studies was acceptable, with no study rated at high risk of bias, supporting the reliability of the findings synthesized in this review.

**Table 2:** Risk of bias assessment across included randomized controlled trials. QoL: quality of life; ERAS: Enhanced Recovery After Surgery; LOS: length of hospital stay

Study (author, year)	Randomization process	Deviations from intended interventions	Missing outcome data	Measurement of outcome	Selection of reported results	Overall risk of bias
Forsmo et al., 2016 [9]	Low risk: random allocation mentioned	Low risk: interventions well adhered to	Low risk: outcomes well reported	Low risk: standard methods used	Low risk: all outcomes reported	Low risk
Pagano et al., 2024 [10]	Some concerns: cluster randomization with potential imbalance	Low risk: well-monitored ERAS implementation	Low risk: large sample, minimal loss	Low risk: objective LOS data	Low risk: protocol registered (NCT04037787)	Some concerns
Lee et al., 2013 [11]	Low risk: 1:1 randomization reported	Some concerns: slight crossover possible, no blinding	Low risk: all patients followed to discharge	Some concerns: subjective outcomes (pain, QoL) not blinded	Low risk: key endpoints reported	Some concerns

Discussion

Past Perspective: Evolution of ERAS in Colorectal Surgery

Historically, perioperative care in colorectal surgery was characterized by prolonged fasting, delayed ambulation, liberal opioid use, and extended hospital stays. These practices often led to increased physiological stress, complications, and higher healthcare costs. The concept of ERAS emerged in the late 1990s, pioneered by Kehlet and colleagues, as a multimodal approach designed to attenuate the surgical stress response and promote faster recovery. ERAS was initially applied in colorectal surgery, integrating elements such as early mobilization, oral feeding, preoperative counseling, and opioid-sparing analgesia. Over the past two decades, ERAS has evolved from a novel protocol to a widely recognized perioperative strategy across various surgical disciplines.

Present Insights: Findings From This Review

This systematic review evaluated three RCTs comparing ERAS protocols with conventional perioperative care in colorectal surgery, with a focus on postoperative morbidity and LOS. Across all included studies, ERAS protocols consistently demonstrated a reduction in LOS. Forsmo et al. [9] reported a significant decrease in median hospital stay from eight days in the control group to five days in the ERAS group (p = 0.001), without an increase in complications, readmissions, or mortality. Similarly, Pagano et al. [10], in a large multicenter stepped-wedge cluster RCT, found that mean LOS declined from 8.5 to 7.5 days following ERAS implementation, with an adjusted reduction of 0.58 days (p = 0.021), again with no significant difference in complication rates. In contrast, Lee et al. [11] observed no statistically significant difference in LOS (7.5 vs. 8.0 days, p = 0.882), although a non-significant trend toward higher postoperative complications was noted in the ERAS group. These findings collectively suggest that ERAS protocols are associated with a clinically meaningful and statistically significant reduction in hospital stay, without compromising patient safety. A detailed summary of these statistical outcomes is provided in Table 3.

**Table 3:** The summary of statistical outcomes comparing ERAS and conventional care across included studies. ERAS: Enhanced Recovery After Surgery; LOS: length of hospital stay

Study	Sample size (ERAS/control)	Mean/median LOS (ERAS vs. control)	p-value for LOS	Complication rate (ERAS vs. control)	p-value for complications
Forsmo et al., 2016 [9]	324	Median: 5 vs. 8 days	0.001	Not significantly different	>0.05
Pagano et al., 2024 [10]	1,337/1,060	Mean: 7.5 vs. 8.5 days	0.021 (adjusted)	OR: 1.22 (95% CI: 0.89–1.68)	NS
Lee et al., 2013 [11]	52/46	Mean: 7.5 vs. 8.0 days	0.882	42.3% vs. 24.0%	0.054 (not significant)

The findings of this review have clear and meaningful implications for clinical practice. The consistent reduction in LOS observed in ERAS groups-without an associated increase in complications-strongly supports the routine adoption of ERAS protocols in colorectal surgery [12]. ERAS achieves these benefits by minimizing the physiological stress of surgery through early ambulation, optimized fluid management, preoperative counseling, and the use of multimodal, opioid-sparing analgesia [13]. These elements work synergistically to accelerate recovery, improve patient comfort, and reduce postoperative complications like ileus or delayed mobilization. From a healthcare systems perspective, implementing ERAS can significantly enhance hospital efficiency by reducing bed occupancy and resource consumption, allowing for faster patient turnover and potentially lowering healthcare costs [14,15]. Consequently, these findings advocate for ERAS protocols to become the standard perioperative care model in colorectal surgery, improving both patient outcomes and institutional workflow.

These findings are generally consistent with prior meta-analyses and systematic reviews that have supported the use of ERAS protocols in colorectal surgery [16]. For example, earlier meta-analyses such as Greco et al. [17] and Gustafsson et al. [18] reported reductions in LOS and postoperative complications with ERAS implementation across surgical fields. However, this review adds value by focusing solely on RCTs, offering updated, trial-level evidence specific to colorectal procedures. Notably, the inclusion of a large-scale stepped-wedge cluster RCT by Pagano et al. [10] reflects a recent shift toward regional-level implementation supported by structured compliance strategies such as audit and feedback, which were not prominent in earlier literature. This evolution highlights an important advancement in the practical application and scalability of ERAS programs [19].

This review’s primary strength lies in its strict inclusion of RCTs, which enhances the validity and reliability of the conclusions. By adhering to PRISMA guidelines and focusing on clinically significant outcomes such as LOS and postoperative morbidity, the review ensures a patient-centered and evidence-based perspective. The included studies also span diverse healthcare systems, which strengthens the generalizability of findings. Nonetheless, the review is not without limitations. The number of eligible RCTs is limited, and there is notable heterogeneity in the specific ERAS elements used across studies, which may impact consistency in outcomes. Additionally, two of the studies had some concerns for bias, particularly regarding measurement and cluster randomization methods, which may influence the strength of the pooled conclusions.

Future Directions: Advancing ERAS Protocols

Despite promising evidence, several gaps remain in the literature that warrant further investigation. Larger, multicenter RCTs with standardized ERAS protocols are needed to reduce heterogeneity and better quantify the independent impact of individual ERAS components. Moreover, future research should extend beyond short-term surgical outcomes to include long-term endpoints such as patient satisfaction, functional recovery, and cost-effectiveness [20]. The integration of ERAS with emerging technologies-such as real-time digital monitoring tools, remote postoperative support systems, and artificial intelligence-driven compliance alerts-may further optimize outcomes and enhance adherence to protocols. Additionally, stratification of outcomes by patient characteristics, including age, comorbidities, nutritional status, and surgical complexity, would help personalize ERAS implementation. Expanding ERAS access to low- and middle-income countries through capacity-building efforts, training programs, and cost-adapted models also represents an essential avenue for future progress. Such initiatives will be vital in refining ERAS protocols and optimizing their global application in colorectal and broader surgical practice.

## Conclusions

This systematic review of three RCTs provides robust evidence that ERAS protocols significantly reduce postoperative LOS without increasing morbidity in patients undergoing colorectal surgery. These findings reinforce the safety and clinical utility of ERAS as a superior alternative to conventional perioperative care. By synthesizing recent, high-quality trial data, our review not only affirms the effectiveness of ERAS but also highlights the growing feasibility of its implementation across varied clinical settings. The importance of this work lies in its focus on rigorous, trial-level evidence to guide surgical practice, offering clear, evidence-based justification for the broader adoption of ERAS pathways in colorectal surgical care.
